# Assessing Consumer Attention and Arousal Using Eye-Tracking Technology in Virtual Retail Environment

**DOI:** 10.3389/fpsyg.2021.665658

**Published:** 2021-08-09

**Authors:** Nayeon Kim, Hyunsoo Lee

**Affiliations:** ^1^Research Center for Future Environmental Design, Institute of Symbiotic Life-TECH, Yonsei University, Seoul, South Korea; ^2^Department of Interior Architecture and Built Environment, College of Human Ecology, Yonsei University, Seoul, South Korea

**Keywords:** eye-tracking, visual attention, emotional arousal, virtual reality, visual merchandising, retail environment

## Abstract

This study aims at investigating how consumers experience the retail environment visually, thus establishing a foundation for deeper insights into visual merchandising strategies. Specifically, we experimentally recorded and analyzed the visual attention and emotional arousal of the consumers in a test setting and examined the influence of various elements as well as gender differences in the recorded consumer responses. We conducted an experiment utilizing eye-tracking and virtual reality to analyze visual attention and emotional arousal in response to spatial and design elements in an immersive retail environment. We examined real-time measures of consumer interest and emotional responses during the retail experience. Valid gaze data from 24 male and 22 female participants were used for the analysis of total dwell time (TDT), total fixation count (TFC), and average pupil diameter (APD). The visual attention and emotional arousal of consumers showed different responses to specific areas of interest according to different spatial arrangements in the sales and service areas. This study statistically analyzed gender differences in consumer responses and performed a correlation analysis between visual attention and emotional arousal. Our findings provide insight into improving the design of retail environments for target consumers and contribute to building visual merchandising strategies.

## Introduction

Consumption patterns and shopping experiences have dramatically changed because of digital transformations. E-commerce is reshaping the shopping behavior of consumers, thus affecting the future of brick-and-mortar businesses (Jocevski et al., 2019; Baek et al., 2020; Roggeveen and Sethuraman, 2020). Although offline retail stores have traditionally focused on driving sales, consumers now prefer the convenience of online shopping (Reinartz et al., 2019). Retailers are, thus, forced to attract consumers by offering them a shopping experience that cannot be replicated online (Jang et al., 2018). New retail environments are now built to attract the attention of consumers by creating unique visual experiences that contribute to consumers staying longer and revisiting the store (Kim, 2020; Kim and Lee, 2020). However, there is a lack of research on consumers' underlying cognition and emotion in retail store environments; additionally, the theoretical foundations for building evidence-based design are limited in marketing studies.

In consumer research, self-report methods such as questionnaires, focus group interviews, and self-assessments are used to understand the psychological reactions of consumers (Ariely and Berns, 2010). The general assumption is that biosensing data can provide marketers with information that cannot be obtained from conventional marketing methods (Khushaba et al., 2013). Further, research has shown that physiological data describing the emotional responses of consumers to functional products are disassociated from self-reported data (Bettiga et al., 2020). Because of the limited technological and analytical capacity, few studies have attempted to empirically analyze how consumer emotion is captured and how they respond to marketing stimuli (Huddleston et al., 2018). Therefore, this study explores the quantification of the underlying consumer attention and emotion beyond the self-report methods.

To understand consumer attention and cognitive process, we investigated the emerging attention-based marketing research that integrates eye-tracking technology and employs the theories and concepts of eye movement control (Orquin and Wedel, 2020). Business success often depends on attracting consumer attention (Davenport and Beck, 2001); within this context, scholars and strategists practice attention-based marketing, drawing insights from consumer behavior, vision research, cognitive psychology, and neuroscience, to optimize marketing efforts (Orquin and Wedel, 2020). Most prior attention-based marketing studies investigated the ways to analyze stimuli, such as products, package designs, advertising, and visual display elements, to determine how attention is correlated with consumer behavior and decision-making (e.g., Seva et al., 2011; Clement et al., 2013; Harwood and Jones, 2014; Huddleston et al., 2015; Deng et al., 2016; Boscolo et al., 2020). This study argues that research on marketing stimuli needs to be extended further to retail environments, such as the elements and features of physical stores.

In general, a large number of studies have focused on capturing eye movement, and few studies have collected pupillary response data. This study further examines the underlying consumer emotion by measuring pupil dilation and investigates correlations between eye movement and pupillary responses. The findings on the correlated visual attention and emotional arousal can provide insight into visual reasoning while experiencing retail environments.

The potential of the eye-tracking method can further reveal gaze information in consumer segmentation. In consumer studies, segmentation represents the different needs of the target market, since age, gender, income, cultural background, and educational levels may be associated with different visual attention and behavior patterns (Dos Santos et al., 2015); further, their emotional responses are processed differently by individuals (Bettiga et al., 2020). Consequently, the use of eye-tracking as a neuromarketing tool can contribute to measure the individual cognitive and behavioral processes related to vision and find the results of attributes and specific patterns commonly found in segmentation criteria (Dos Santos et al., 2015). In general, previous studies are limited to measuring the average eye movements of all participants. However, unlike most of the previous studies, focused on analyzing averaged fixation data, this study tackles individual differences, such as different gender groups, to compare how the male and female subjects visually respond to the retail environments. It is necessary to understand the effects of individual gaze characteristics and analyze visual reasoning and preferences of target consumers such as gender differences (Kim, 2020).

Our goal is to improve the understanding of how consumers visually experience retail store environments and generate evidence and insights on improving store atmosphere to contribute to building visual merchandising strategies for target consumers. The main objectives of the present study are: (1) to assess the influence of different elements in a store environment on consumer responses, (2) to examine gender differences in visual attention and emotional arousal, and (3) to analyze correlations between visual attention and emotional arousal.

To achieve our objectives, we employ eye-tracking technology to reflect the cognitive and emotional responses of participants by observing their eye movement patterns and gaze characteristics during simulated retail experiences in virtual environments. We aimed to quantify the unconscious arousal and attention of participants using a biosensor and a virtual reality (VR) system. We assume that higher total dwell time (TDT) values and fixation counts represent higher visual attention, and greater pupil dilation represents higher arousal. We expect the findings of this study to help retailers create better retail environments to attract and retain consumer attention. Our results can be used to integrate the design of retail environments with visual merchandising strategies.

## Literature Review

### Eye-Tracking and Virtual Environment

Neuroscience research has been employed widely in design fields to understand the responses of consumers, and physiological measures have been used to assess the experiences of consumers with new products (Bettiga et al., 2020). Consumer neuroscience can be utilized especially to link consumer choice and decision-making to marketing research (Camerer et al., 2004; Pirouz, 2007; Plassmann et al., 2012; Khushaba et al., 2013). The use of eye-tracking as a neuromarketing tool has potential in practical marketing applications, such as brand equity, segmentation, new product development, and social marketing studies (Dos Santos et al., 2015). Technological developments in eye-tracking methods make it possible to measure consumer attention during shopping experiences (Meißner et al., 2019).

As a physiological measurement, scholars have proposed the use of eye movement information (Bednarik et al., 2005; Bergstrom and Schall, 2014). Eye-tracking devices vary from screen-based applications to mobile glasses and VR headsets and can record the eye movements of participants in real time (Borgianni and Maccioni, 2020), thus offering insights into what people may be interested in (Goldberg and Kotval, 1999). Experiments using eye-tracking behavior have been widely conducted in laboratory and field settings to examine user experiences (Bergstrom and Schall, 2014; Kim and Lee, 2020). A previous study proposed the use of eye movement as a biometric measurement (Bednarik et al., 2005). The placement of visual attention and psychological reactions to changing conditions are related to the cognitive state (Kiefer et al., 2017).

The eye-tracking methodology tracks the gaze and eye movements of consumers; it can be used to identify points of interest and pinpoint sources of information (Holmqvist, 2011). The eye-tracking methodology is used to evaluate design attributes and visibility and has been employed in usability research in the field of human-computer interaction since the 1990s (Tonbuloǧlu, 2013). In design studies, this methodology has been adopted to understand design reasoning, creativity, and design analysis (Matthiesen et al., 2013; Yu and Gero, 2016; Self, 2019; Gero and Milovanovic, 2020). It also contributes to environmental design because it can provide architects and designers with more precise insights on how the design affects the behaviors and well-being of occupants (de Paiva, 2018).

In consumer research, eye-tracking experiments have been conducted to assess marketing stimuli, such as product packaging and point-of-purchase marketing (Huddleston et al., 2015). These studies examined which stimuli and areas consumers focused on the most and analyzed the consumer search-choice process in retail environments through eye-tracking experiments (Huddleston et al., 2018).

Consumer and neuromarketing researchers have employed VR-integrated eye-tracking systems (Clay et al., 2019) since 2017. Virtual reality simulations can potentially increase the value of retail research and make it possible to test different environments in the same geographical location. Virtual reality in marketing is a very promising technological tool that can help provide satisfactory consumer experiences that mirror those experienced in brick-and-mortar stores (Alcañiz et al., 2019). Duchowski et al. (2000) have noted that while VR-integrated eye-tracking systems are relatively new, they are a promising development. However, in general, few studies have been conducted in this emerging field of research.

In the relevant literature, immersive VR stimuli are expected to produce more representative experimental data. Virtual reality technologies can provide interactive, immersive, and realistic experiences with little investment (Tovares et al., 2014). Eye-tracking systems can be seamlessly integrated with VR systems for greater applicability in research (Jacob and Karn, 2003). This study explores how users react to environmental stimuli by measuring the attention and arousal of participants while they are experiencing an immersive virtual setting.

### Measuring Visual Attention and Emotional Arousal

Eye-tracking devices and software can capture eye movement and pupil dilation (Gero and Milovanovic, 2020) and collect vision-related data points, such as gaze coordinates, fixation duration, number of fixation points, gaze path, gaze pursuit, gaze velocity, pupil size, and pupil dynamics (Bednarik et al., 2005). Relatively steady eye movements represent fixation on a particular point or scanning of a particular area. Thus, information about gaze fixation and duration can reflect cognitive states (Jacob and Karn, 2003). The raw data can then be used to map the eye movements of participants and visualize them in the form of attention maps, such as heat maps (Holmqvist, 2011). Such visual attention data can be subjected to group- and individual-based analyses to identify variables that affect the experiences of participants (Kim and Lee, 2020).

Eye-tracking devices allow researchers to record, in real time, where a user looks and for how long, and to measure pupil diameter. Emotional states can also be investigated using biometrics. Pupil dilation reflects psychological excitement; therefore, internal processes can be analyzed by measuring changes in pupil size (Kahneman, 2011). Pupil size often changes in response to emotional and sensory reactions in humans, and therefore, it is helpful in identifying the psychological states of consumers. Emotional intensity and strength of stimulation are positively correlated with the degree of pupil dilation Janisse (1974). The stronger the stimulation, the greater the pupil dilation (Nunnally, 1994).

A previous research analyzed the pupils of participants to evaluate their emotional responses in a built environment (Kim, 2018). Thus, in this study, pupil dilation is measured as a proxy for emotional arousal. By using biometrics, this study overcomes the limitations of self-report methods that were traditionally used to understand the emotional states of consumers. Our measurement of visual attention and emotional arousal is based on previous eye-tracking studies (Holmqvist, 2011; Bergstrom and Schall, 2014).

### Visual Merchandising in a Retail Environment

The retail environment itself is a combination of the physical and the emotional, that is, tangible and intangible attributes (McGoldrick, 2002; Varley and Rafiq, 2004; Barnes and Lea-Greenwood, 2010). Such environments are thus designed to produce conscious and unconscious effects on the purchasing decisions of consumers (Jang et al., 2018). Their interactions with retail environments influence their emotional states and shopping behavior (Baker et al., 1992; Donovan et al., 1994; Spies et al., 1997; Mummalaneni, 2005).

A retail marketing strategy includes visual merchandising, whereby a retailer creates an in-store environment to visually communicate with consumers and encourage them to make a purchase (Pegler, 2012). Visual merchandising makes products more attractive to the target consumers (Soomro et al., 2017). Additionally, in-store environmental attributes, such as overall atmosphere, store layout, design and display elements, and background music, trigger emotional states in consumers and lead to behavioral responses, such as intention to purchase (Matthews et al., 2013; Wanniachchi and Kumara, 2016; Venter de Villiers et al., 2018).

The perception of visual merchandising produces affective responses during the experiences of consumers (Law et al., 2012). Thus, positive consumer emotional responses to visual merchandising are causally related to positive brand image and help drive up sales. Accordingly, understanding how consumers direct their visual attention to merchandising could help create better brand experiences in retail environments.

Physical, functional, and emotional aspects of design elements must be considered when engaging in visual merchandising. In a retail environment, visual merchandising elements include a variety of factors, such as store layout and design, window display, color combination, logo signage, lighting, product placement, mannequin placement, furniture and fixtures, and prop selection (Soomro et al., 2017). Visual merchandising elements are categorized into three factors: environmental, display, and communication (Lee Han, 2013). Environmental factors include the façade of the store, building structure, architectural design and materials, lighting, sound, and scent. Display factors for sales and service refer to wall fixtures and table fixtures for item presentation, fitting rooms, checkout counters, service lounge seating, mannequins, and props. Communication factors include logo signage, point-of-purchase displays, catalogs, and employee uniforms. Our study considered the three factors mentioned above to analyze elements in retail environment.

## Materials and Methods

The study examines how the elements of the retail environment affect the responses of the individual consumer using quantitative research methods. The research was mainly conducted using eye-tracking technology in a VR setting. The visual stimuli were developed to examine different conditions by spatial arrangement in a retail store environment.

### Participants

The participants were recruited from University campuses, office buildings, and residential buildings located in Seoul, South Korea. We employed a voluntary sampling method using an online registration system, which allowed us to recruit potential participants and schedule the visits of individual participants to the experiment lab. The recruitment advertisement was posted on an online platform. Subsequently, those who were target consumers for the brand of the case and those interested in participating in the experiment could sign up for an available date and time. Each potential participant could only participate in one experiment.

This study was conducted with 50 participants, all aged between 21 and 35 years and included 25 males and 25 females. For accuracy and reliability, gaze data for analysis were collected only from participants who recorded a tracking ratio of over 85%. Valid gaze data from 24 male and 22 female subjects were captured. All methods and experimental protocols were approved by the Institutional Review Board of Yonsei University, and all participants signed a consent form prior to the experiment.

### Stimuli

**Figure 1:**
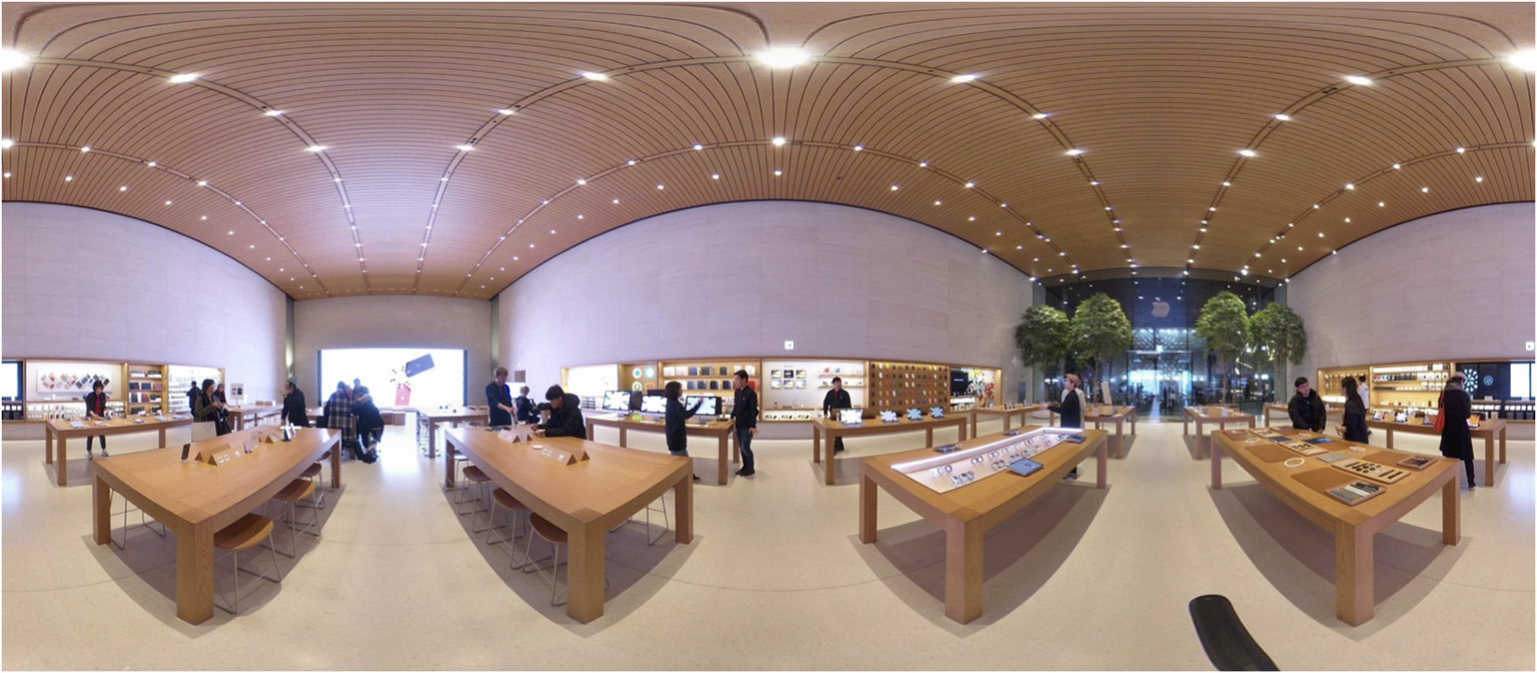
360° panoramic image of Stimulus 1.

**Figure 2:**
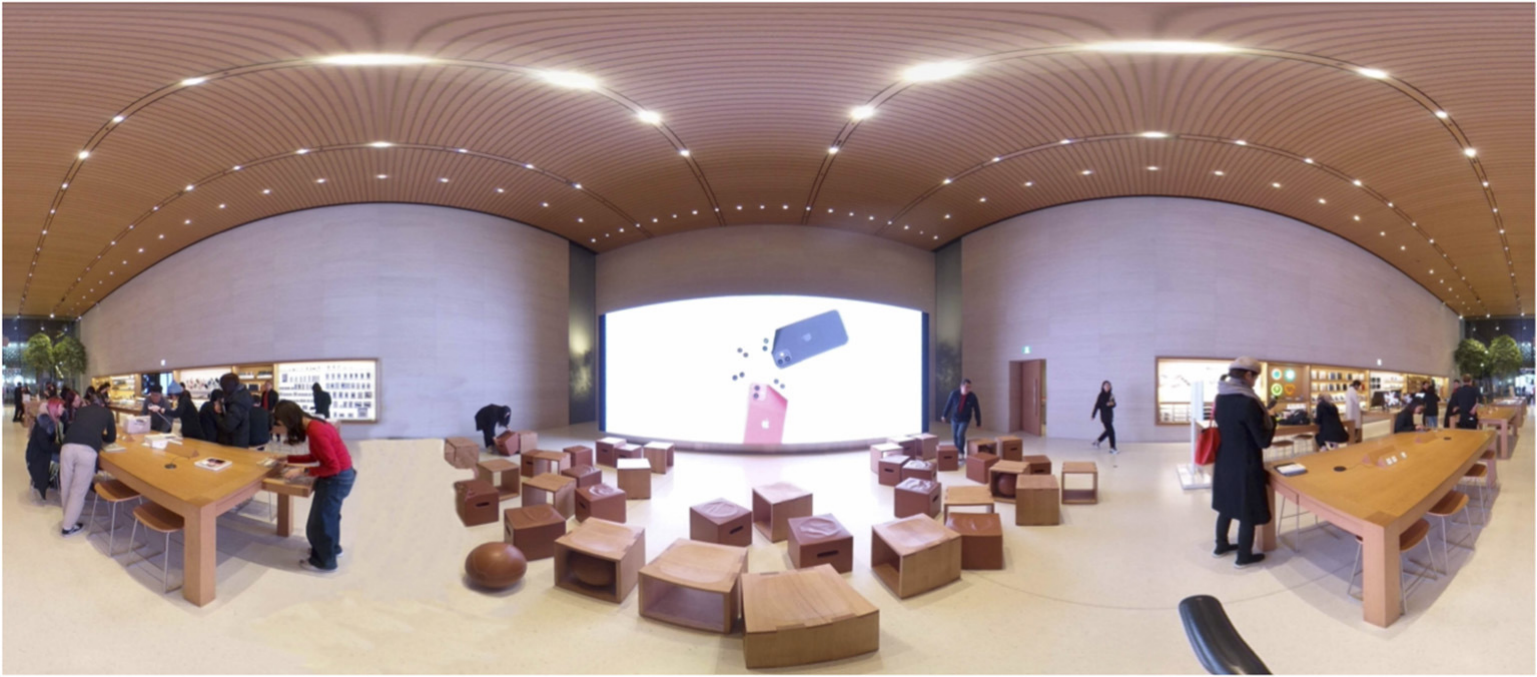
360° panoramic image of Stimulus 2.

**Figure 3:**
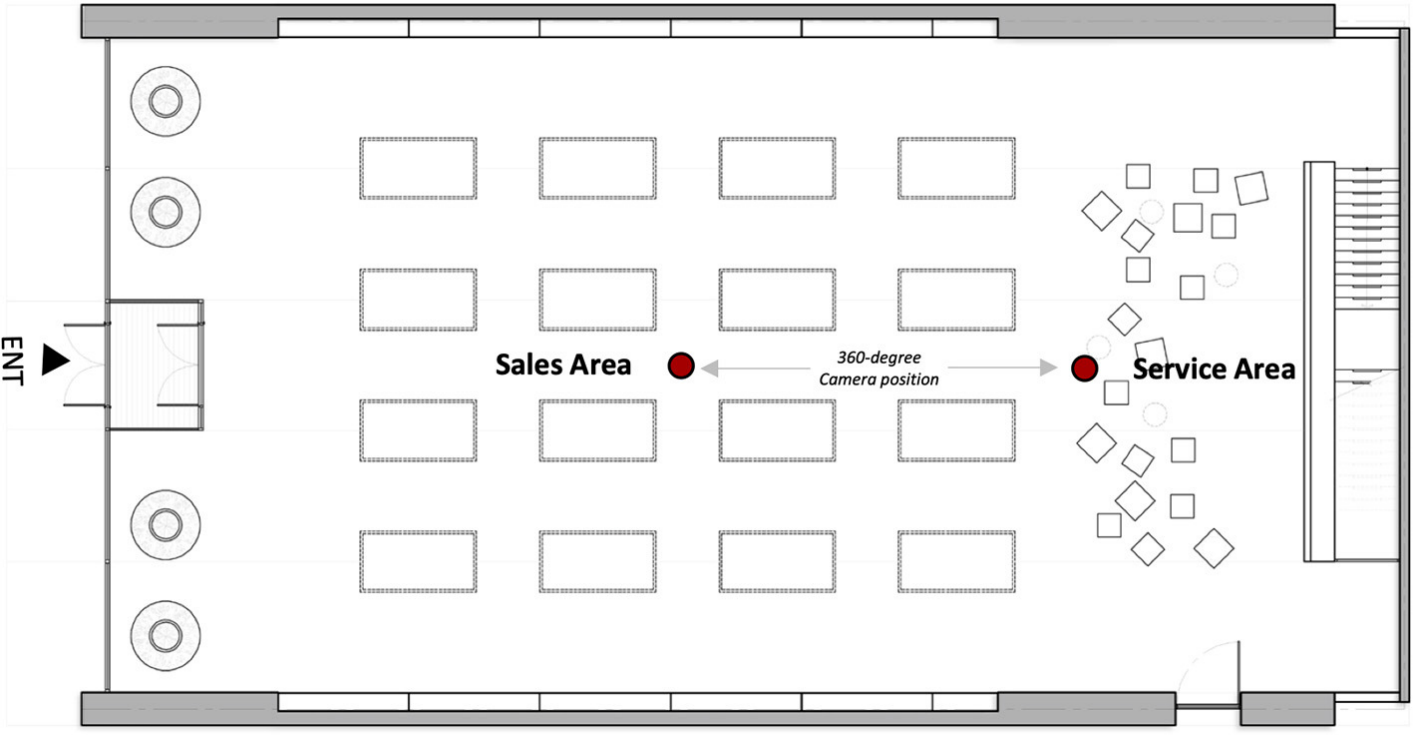
Floor plan.

The visual stimuli were developed and simulated for use in a head-mounted VR display. Still images from a 360° camera were created with a “Theta V” by RICHO, which can capture natural 360° still images, video content with a high resolution, and perform highly precise image stitching to process images into a unified panorama. The original retail environments were initially modeled from a camera that recorded 360° panoramic videos and photos. For a comparison of different virtual retail environments, each image presented a sales area and a service area by spatial function. This study was designed to understand how people respond differently to different conditions in retail store environments in terms of sales vs. service areas. Using Adobe Photoshop, the final selection of images was modified and used as experimental stimuli. Figure 1 refers to Stimulus 1 and includes the sales and display area. Figure 2 refers to Stimulus 2, which represents the service areas, including video wall, forum, and support. After the site survey, the floor plans were drawn using AutoCAD. Figure 3 shows the floor plan for a retail store environment.

### Experimental Procedure

**Figure 4:**
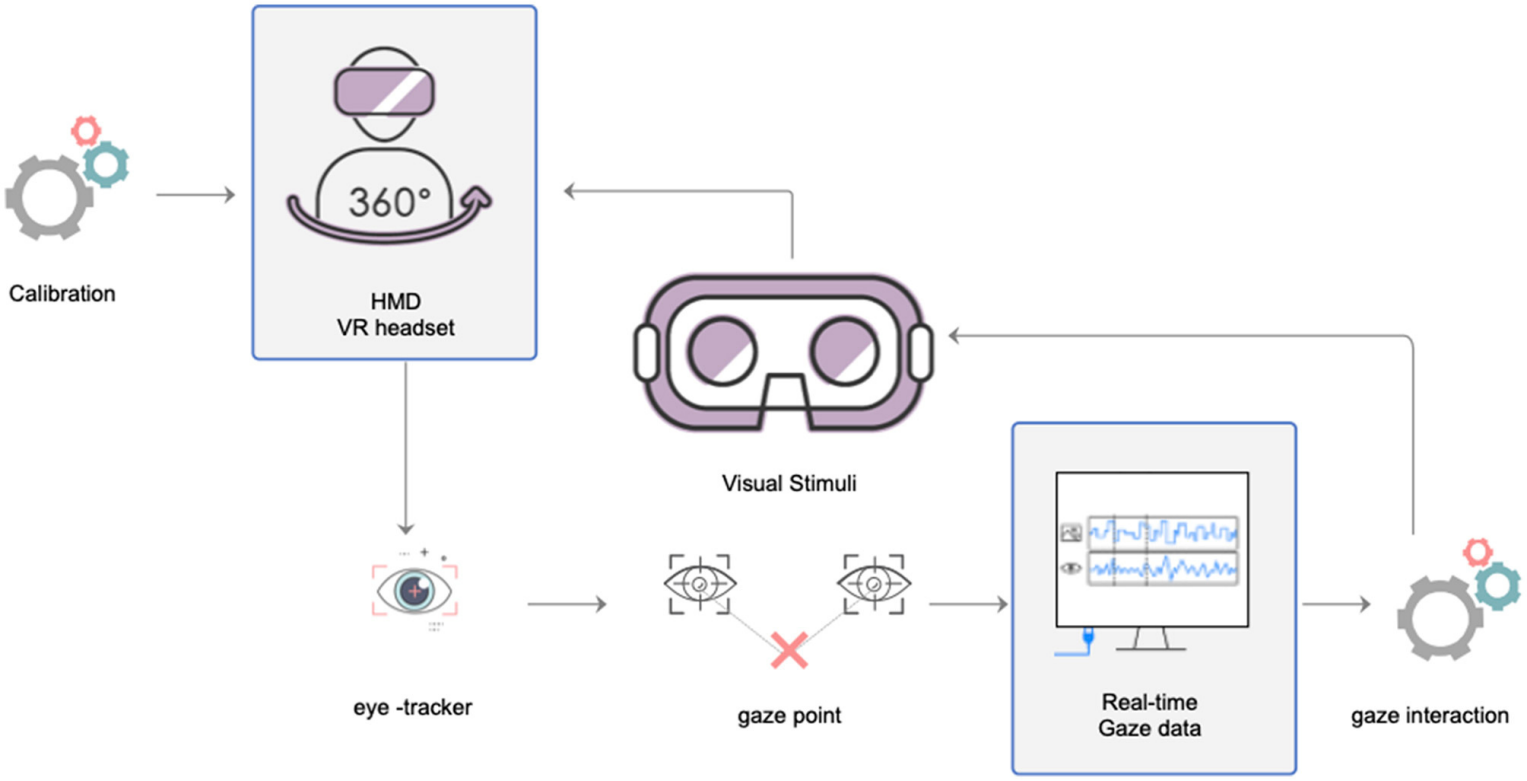
Mechanism for VR system with eye-tracking.

Virtual reality-embedded eye-tracking systems include both hardware and software components. Clay et al. (2019) described the hardware and software components of typical systems, as well as resources and tools, from a variety of manufacturers. A laptop with the necessary software preinstalled was set up in an experimental lab to serve as an interface between the eye-tracker and VR hardware and to collect data on the behaviors of participants (Clay et al., 2019). Figure 4 shows the mechanism of the VR system with eye-tracking. This diagram was modified from the gaze-based interaction scheme for VR environments given by Piotrowski and Nowosielski (2019).

The hardware used in this experiment is the HTC-Vive, manufactured by Sensor Motoric Instruments (SMI) in Germany. The integration of biosignal sensors in the HTC-Vive device enables controlled environments for naturalistic eye-tracking studies. To collect data from all the subjects during the experiment, software for the SMI experiment suite for stimulus analysis, such as the C/C++ Software Development Kit, Experiment Center, BeGaze, Steam VR, and Unity 3D, was installed on the laptop. This HMD VR device enables the real-time capture of user gaze data during the VR experience. By simply setting start and end times for the experiments, the raw eye data of the user are synchronized with the time when the actual stimuli are presented.

**Figure 5:**
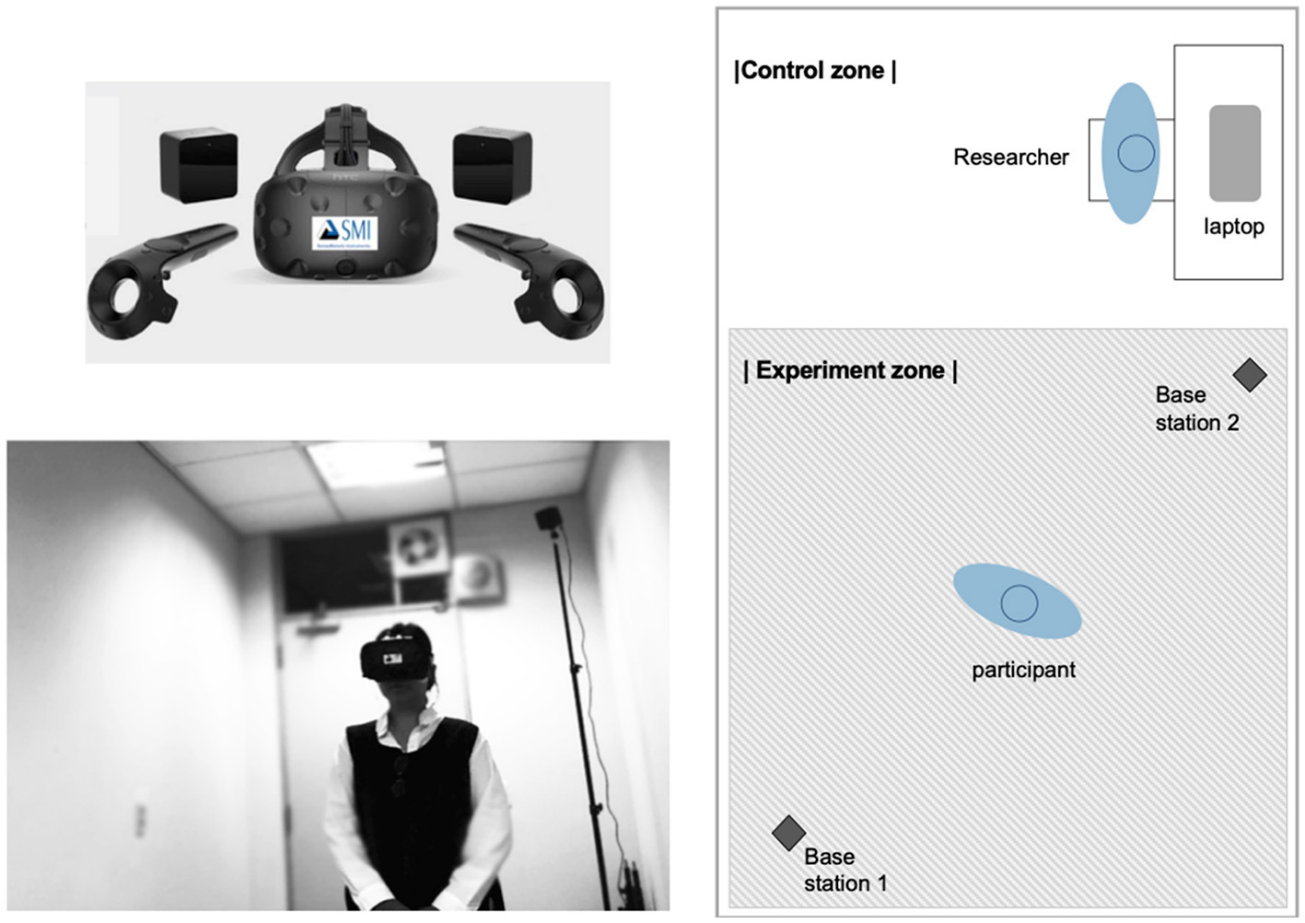
Materials and experiment setup.

During the experiments, gaze data were automatically transmitted from the HMD device and displayed on the laptop screen in real time. This allowed a researcher to check the gaze data of each user and manage his/her eye-tracking status and the progress of his/her session simultaneously. In this study, two cubical base stations were placed in the experimental lab to define the boundaries of the virtual space, outside of which the VR headset would not function. Figure 5 shows the materials for the VR experiment setup in the laboratory of the university.

In the procedure of the main experiment, each data collection process was conducted individually with a researcher and took approximately 30 min. As soon as a participant arrived at the experiment lab, the researcher explained the overall research procedure to the participant. All subjects were carefully informed of the step-by-step precautions of the experiment. In the experiment lab, the subjects wore an HMD device with an integrated eye-tracking function. Each participant was taken through a calibration procedure prior to the observation, after which the observation was conducted under the control of the researcher.

During the experiment, each participant was asked, while wearing the HMD device, to view two panoramic images of a 360° virtual retail space. All participants were exposed to visual stimuli in sales and service areas, the order of which was randomly assigned. Each visual stimulus was shown for 30 s to collect the eye-tracking data and visual attention patterns of participants for each image. All participants were exposed to visual stimuli in 2 types of spaces. Eye-tracking data were collected with a 250 Hz binocular.

### Eye Movement Metrics

To examine the effects of elements in-store environment on consumer responses, we defined and classified each major element as an area of interest (AOI), where AOIs refer to regions of display that the researchers define and classify by shape and for which quantitative data can be calculated (Bergstrom and Schall, 2014). In this study, AOIs are spatial and visual elements in the retail store environment. In total, we identified 10 AOIs; AOI elements were categorized into three groups: environmental factors (e.g., façade, floor, wall, and ceiling), display factors, (e.g., wall fixture, display table, seating, and props), and communication factors (e.g., logo signage and media screens).

Based on the key performance indicators of eye movement, we focused on eye-tracking metrics to observe the visual attention of participants to AOIs: TDT, total fixation count (TFC), and average pupil diameter (APD). Total dwell time and fixation counts generally indicate that the visual attention of a participant was attracted to a particular AOI (Resnick and Albert, 2014; Hwang and Lee, 2018). Fixations indicate elements or areas where information collection and processing can occur (Kim and Kim, 2019). Fixation count is also a measure of the importance of the visual attention (Jacob and Karn, 2003).

First, we measured the TDT on AOIs. The total fixation time is the amount of time participants spent looking at a particular AOI. Second, we calculated the number of fixations on AOIs. A higher fixation count indicates that specific elements or areas are more important and interesting (Jacob and Karn, 2003) than others. Lastly, we measured the average pupil diameter to understand emotional arousal when they see a specific AOI.

## Analysis and Results

The average gaze data collected from the participants were analyzed to measure the visual attention and emotional arousal responses to AOIs in Stimulus 1 and Stimulus 2. Higher TDT and TFC referred to higher visual attention, and a higher value for APD indicated higher emotional arousal to the AOI.

Statistical analyses were carried out using SPSS (Version 25.0). Descriptive statistics presented sample size, maximum and minimum values of the variables, means, and standard deviation (*SD*). We utilized the *t*-test to judge whether there existed significant differences in gaze parameters between male and female participants; degree of freedom, *t*-statistics, and *p*-values were also reported. A *p*-value <0.05 was considered statistically significant.

### Visual Attention

#### Distribution of Visual Attention for AOIs

**Figure 6:**
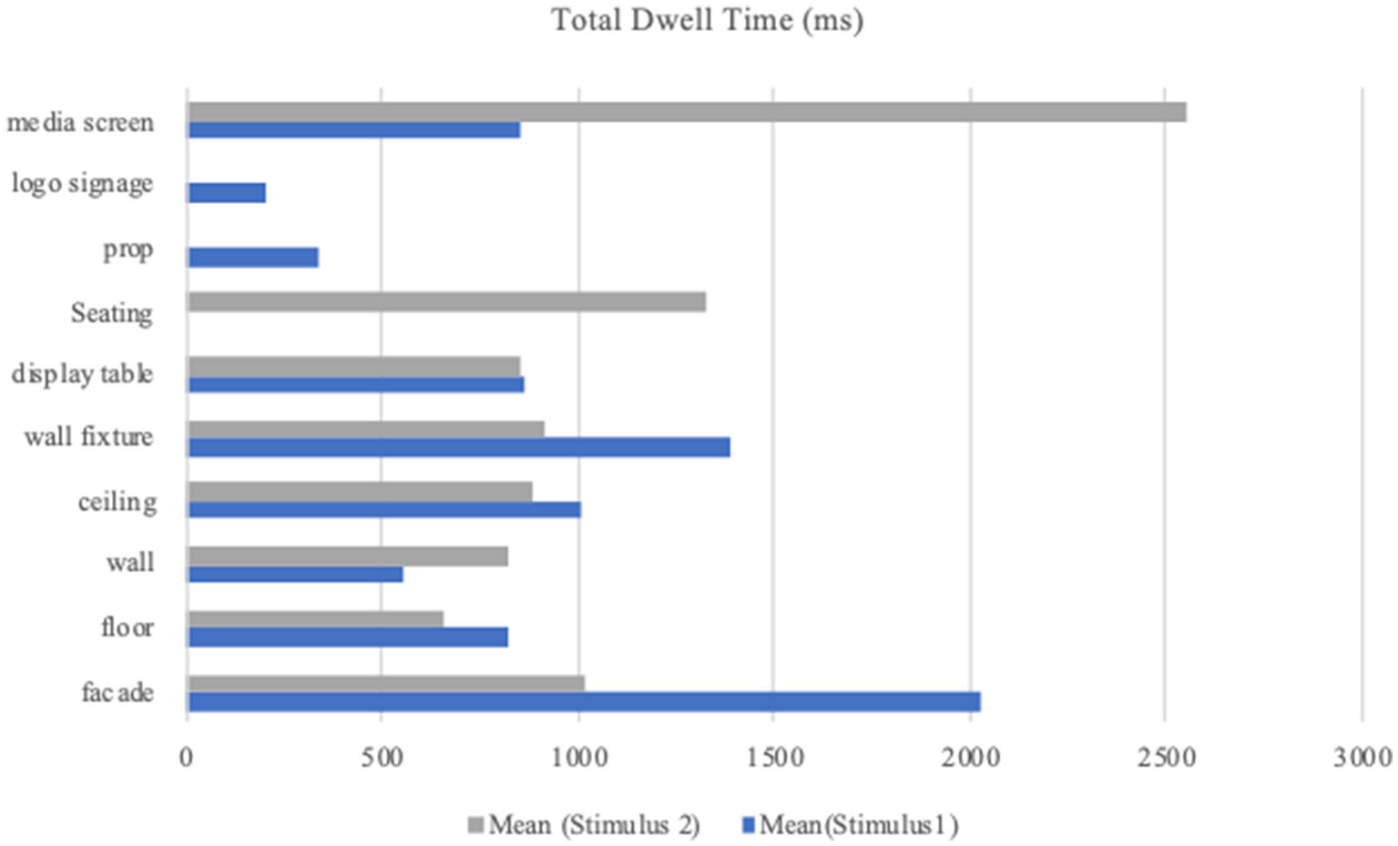
Mean value of total dwell time per AOI.

In this study, the statistical data related to Stimulus 1 demonstrate that participants did not fixate on the seating. Among the other nine AOIs (excluding seating), the mean value was the highest for the façade (*M* = 2,025.23; *SD* = 1,278.82) and the lowest for the logo signage (*M* = 207.98; *SD* = 174.38). The mean value for the other AOIs ranged from 338.76 (prop) to 1,384.36 (wall fixture). In Stimulus 2, the data results demonstrate that there was no fixation on prop and logo signage. Among the other eight AOIs (excluding props and logo signage), the mean value was the highest for the media screen (*M* = 2,552.77; *SD* = 2,087.11) and the lowest for the floor (*M* = 654.45; *SD* = 731.86). The mean values for the other AOIs ranged from 818.85 (wall) to 1,323.66 (seating). Table 1 presents the means and SDs for TDT per AOI. Figure 6 presents the mean values for TDT per AOI in comparison with Stimulus 1 and Stimulus 2.

**Table 1:** Means and standard deviations for total dwell time per AOI.

**AOI**	**Stimulus 1 (TDT)**			**Stimulus 2 (TDT)**		
	***n***	**Mean**	***SD***	***n***	**Mean**	***SD***
Facade	42	2,025.23	1,278.82	8	1,018.53	848.72
Floor	25	816.89	849.79	23	654.45	731.86
Wall	41	556.41	389.83	44	818.85	615.60
Ceiling	25	1,004.81	1,252.46	19	884.44	906.07
Wall fixture	45	1,384.36	688.26	39	916.06	524.76
Display table	44	864.69	628.90	33	853.90	825.98
Seating	1	0.00	0.00	36	1,323.66	1,163.63
Prop	29	338.76	288.74	0	0.00	0.00
Logo signage	6	207.98	174.38	0	0.00	0.00
Media screen	33	848.47	720.66	41	2,552.77	2,087.11

*AOI, area of interest; TDT, total dwell time. Unit: ms*.

**Figure 7:**
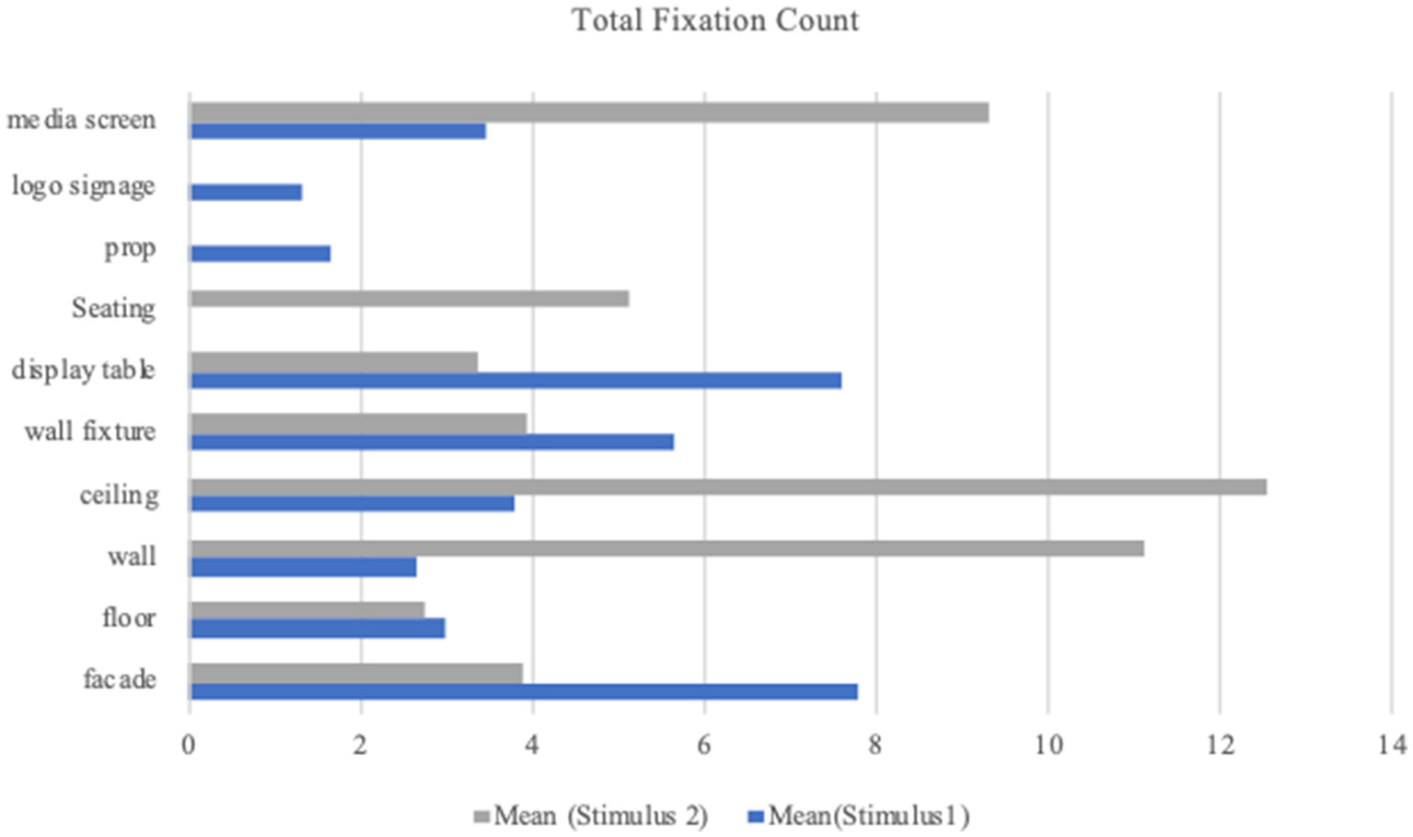
Mean value of total fixation count per AOI.

The TFC was analyzed per AOI for each stimulus. In Stimulus 1, as the gaze fixation of participants had not occurred on seating, the mean value was the highest for the facade (*M* = 7.79; *SD* = 4.40) and the lowest for logo signage (*M* = 1.33; *SD* = 0.51). The mean values for the other AOIs ranged from 1.67 (prop) to 7.59 (display table). In Stimulus 2, there was no fixation on props and logo signage. The highest mean value of TFC across the other AOIs was for the ceiling (*M* = 12.55; *SD* = 38.19) and the lowest was for the floor (*M* = 2.74, *SD* = 2.12). Among the remaining six AOIs, the mean value ranged from 3.34 (display table) to 11.12 (wall). Table 2 presents means and *SD*s for TFC per AOI. Figure 7 shows the mean value of TFC per AOI in comparison with Stimulus 1 and Stimulus 2.

**Table 2:** Means and *SD*s for total fixation count per AOI.

**AOI**	**Stimulus 1 (TFC)**			**Stimulus 2 (TFC)**		
	***n***	**Mean**	***SD***	***n***	**Mean**	***SD***
Facade	42	7.79	4.40	8	3.88	3.14
Floor	24	3	2.95	23	2.74	2.12
Wall	41	2.66	1.58	44	11.12	39.28
Ceiling	25	3.80	4.32	20	12.55	38.19
Wall fixture	45	5.65	2.64	39	3.94	1.90
Display table	44	7.59	27.01	33	3.34	2.46
Seating	1	0	0	36	5.13	3.72
Prop	29	1.67	1.03	0	0.00	0.00
Logo signage	6	1.33	0.516	0	0.00	0.00
Media screen	33	3.48	2.25	41	9.32	6.92

*TFC, total fixation count. Unit: number*.

#### Gender Differences in Visual Attention

As the results of *t*-test for comparing gender in Stimulus 1, the TDT for the media screen was significantly different (*t* = 2.119; *p* < 0.05). The mean values for male and female participants were 1,138.66 and 606.64 ms, respectively. The male participants showed more interest than the female participants in the media screen. The TFC for logo signage was significantly different (*t* = 3.395; *p* < 0.05). The mean values for male and female participants were 401.95 and 111.00 ms, respectively. Thus, the interest of male participants was higher than that of the female participants in the logo signage.

In Stimulus 2, the *t*-test results showed that there was a significant difference between the male and female participants for media screen: TDT (*t* = 3.941; *p* < 0.05) and TFC (*t* = 3.481; *p* < 0.05). The mean value for TDT of male participants was 3,535.67, and for female participants, it was 1,414.66. The TFC was 12.41 in male participants and 5.74 in female participants. Thus, the male participants paid greater attention to the media screen than the female participants.

### Emotional Arousal

#### Distribution of Emotional Arousal for AOIs

In Stimulus 1, across all AOIs, all the pupillary responses of the participant were poor for logo signage and seating. Among the remaining eight AOIs, the mean value was the highest for the prop (*M* = 4.07; *SD* = 0.63) and lowest for the wall (*M* = 3.80; *SD* = 0.61), ceiling (*M* = 3.80; *SD* = 0.85), and display table (*M* = 3.80, *SD* = 0.67). The mean values for the other AOI ranged from 3.84 (wall fixture) to 4.00 (façade).

**Figure 8:**
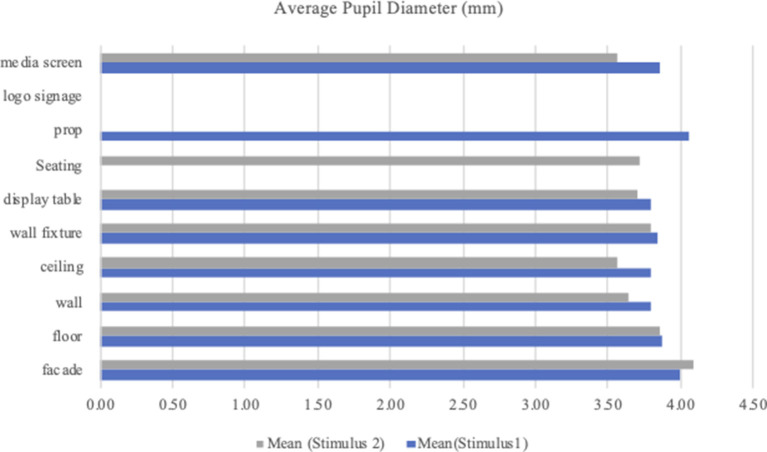
Mean value of average pupil diameter per AOI.

In Stimulus 2, there was no fixation on two AOIs, namely, prop and logo signage, and therefore, they were excluded for analysis of APD. Across the remaining eight AOIs, the highest value for APD was for facade (*M* = 4.43; *SD* = 1.43) and the lowest was ceiling (*M* = 3.60; *SD* = 0.93). The mean value for the rest ranged from 3.63 (media screen) to 3.86 (floor). Table 3 presents the means and *SD*s for the APD per AOI. Figure 8 shows the mean value of the APD per AOI for Stimulus 1 and Stimulus 2.

**Table 3:** Means and *SD*s for average pupil diameter per AOI.

**AOI**	**Stimulus 1 (APD)**			**Stimulus 2 (APD)**		
	***n***	**Mean**	***SD***	***n***	**Mean**	***SD***
Facade	39	4.00	0.56	7	4.32	1.43
Floor	26	3.87	0.61	28	3.86	0.51
Wall	40	3.80	0.61	41	3.73	0.56
Ceiling	24	3.80	0.85	23	3.60	0.93
Wall fixture	42	3.84	0.61	38	3.83	0.69
Display table	41	3.80	0.67	33	3.70	0.56
Seating	0	0.00	0.00	37	3.74	0.65
Prop	33	4.07	0.63	0	0.00	0.00
Logo signage	0	0.00	0.00	0	0.00	0.00
Media screen	31	3.85	0.61	39	3.63	0.54

*APD, average pupil diameter. Unit: mm*.

#### Gender Differences in Emotional Arousal

In Stimulus 1, the *t*-test was used to compare gender by calculating APD to AOI. The result of *t*-test showed that the gender differences for facade was significantly different (*t* = −2.424; *p* < 0.05). The female participants (*M* = 4.22 mm) were more emotionally aroused than the male participants (*M* = 3.81 mm) when their eyes fixated on the façade. There was a significant gender difference in floor (*t* = −2.173; *p* < 0.05) and prop (*t* = −2.353; *p* < 0.05). The results presented that the pupil diameter of the female participants was larger than that of the male participants on floor and prop. Thus, female participants tend to be more interested in façade, floor, and prop in Stimulus 1. However, in Stimulus 2, the *t*-test results showed that there was no significant difference between male and female participants.

### Correlation Analysis Between Visual Attention and Emotional Arousal

Correlations were analyzed to assess the linear relationship between two variables. This study examined the correlation between two variables (i.e., TDT and TFC) of visual attention and one variable (APD) of emotional arousal. The results of the correlation analysis for Stimulus 1 showed that there were no correlations between visual attention and emotional arousal on AOI.

**Figure 9:**
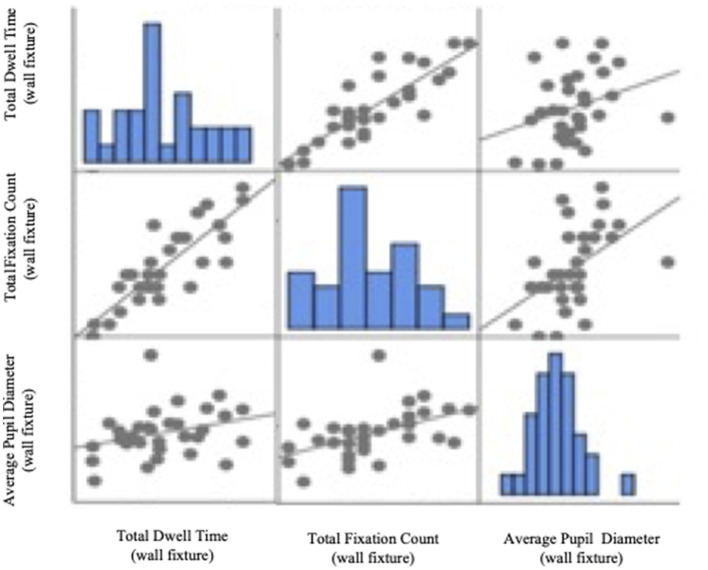
Scatter plots of correlation analysis between visual attention and emotional arousal.

In Stimulus 2, the TFC was positively correlated with APD responding to wall fixture (*p* < 0.01), and its value of Pearson's correlation coefficient is 0.491. The results presented a correlation matrix (Table 4) and a plot (Figure 9). Thus, in the service area, visual attention was correlated with emotional arousal for wall fixture.

**Table 4:** Correlation matrix.

		**Wall fixture (TDT)**	**Wall fixture (TFC)**	**Wall fixture (APD)**
Wall fixture (TDT)	Pearson's *r*	1	0.856**	0.297
	*p*-value	–	0	0.105
Wall fixture (TFC)	Pearson's *r*	0.875**	1	0.491**
	*p*-value	0.000	–	0.005
Wall fixture (APD)	Pearson's *r*	0.297	0.491**	1
	*p*-value	0.105	0.005	–

^*^p < 0.05,

** *p < 0.01*.

## Discussions and Conclusions

### Consumer Response to Different Conditions in the Retail Store Environment

According to the different conditions of spatial arrangements, whether it was mainly focused on the sales and display area (Stimulus 1) or the service area (Stimulus 2) including media screen wall, forum, and support, the visual attention and emotional arousal of the consumer showed different responses to the spatial and design elements in a store environment. The general results of the comparison based on three groups of factors (environmental, display, and communication) in the sales area showed that most participants were visually attracted by environmental factors (e.g., facade, ceiling) and display factors (e.g., wall fixture and display table) in the sales area. In contrast, the results of the comparison based on the three groups of factors showed that most participants were visually attracted by the communication factor (e.g., media screen) in the service area. The participants who demonstrated high attention to the media screen were also interested in looking at the seating area near the media screen. The combination of media screen and freely arranged stools had the most visual impact on participants.

In the sales area, the participants paid more attention to the display table and wall fixture than those in the service area. The findings showed that their gaze remained on the items for a longer period because of their interest in the branded products displayed on top of the table, not because of the aesthetic design attributes of the table fixture itself. The table fixture for sales had a very ordinary rectangular design made of a wooden material, and the attributes of the table fixture itself were not unique or attractive. Thus, the average participants paid the most attention to experiencing the new product displays. The results of TDT indicated that participants looked at the logo signage for the shortest time because only one logo signage was installed on the glass façade of the building; no additional logos were placed in the interior space. To increase the visibility of the brand logo, therefore, we suggest installing a logo signage on the focal wall of the interior space, where it can enhance consumers' brand engagement with their shopping experiences.

In the service area, most participants paid a high level of attention to the large media screen installed on the central wall. It functioned as a focal design element and provided advertising for the latest brand products and promotions. Participants also showed interest in the seating area in front of the media screen, where consumers could freely sit and interact with the staff. This area also displayed advertising for the latest brand products and promotions. Consumers could watch an advertisement video on the media screen while waiting to make a purchase. In contrast, the results showed that the plain wall did not attract the visual attention of participants. Our findings supported the results of Sussman and Ward (2017) that blank or featureless facades do not draw attention, while buildings with high contrast or punched windows do attract attention.

The findings from the results of correlation analysis in the sales area showed that there were no correlations between visual attention and emotional arousal on the AOIs. In the service area, the visual attention was correlated to emotional arousal in the case of wall fixture. However, the specific elements that attracted attention do not always correspond with emotional arousal. Consequently, the results showed that the effects of store environment elements intended to draw visual attention and emotional arousal were partially different according to individual differences.

### Gender Differences in Visual Attention and Emotional Arousal

In the sales area, we found that male participants demonstrated more interest in the media screen and logo signage than the female participants. However, female participants showed more emotional arousal than the male participants in the areas of façade, floor, and prop. In the service area, male participants paid greater attention to the media screen than the female participants.

The findings can provide evidence on how to attract male and female consumers, visually or emotionally, when designing retail store environments. For example, male consumers tend to be attracted by media screens and logo signage on the façade. This indicates that the storefront design should reflect the interactive media façade, which can be one design strategy to attract the attention of male consumers. Our results also show the distinctive interests of gender groups by their responses to visual objects: male participants paid greater attention to wall fixtures, whereas female participants were more interested in display tables and prop. The female participants were also visually attracted by the large-sized plant box used as a prop near entrance areas. The findings demonstrate useful information on where to display new products to sell to the target gender. Male participants were more interested in the media screen on the wall and found it informative and stimulating. They also were highly interested in the cube-shaped seating stools in front of the media screen, indicating that the free arrangement of seating could contribute creatively to brand image. We also suggest incorporating biophilic design principles as a visual merchandising strategy to attract more female customers in the retail environment.

Previous studies in retail marketing were limited to analyzing the average eye movements of all the participants (Huddleston et al., 2015, 2018). However, this study examined individual differences and analyzed the variations between male and female participants. Not only gender differences but also segmentation by age, income, and cultural background in the target market (Dos Santos et al., 2015) needs to be investigated to understand differences in the attention and emotions of their consumers. The findings can be used to develop guidelines for designers to improve the attractiveness of retail environments and reflect the needs and interests of a potential customer.

## Conclusion

An empirical study was conducted to understand the visual attention and emotional arousal of the consumers in a retail store environment during a virtual experience. We conducted eye-tracking experiments to observe the underlying responses of consumers to visual elements under different conditions in a retail environment and assessed how their level of attention and arousal differed by the spatial arrangement of sales and service areas. Visual attention was measured by two indicators: TDT and TFC. Emotional arousal was interpreted from changes measured in APD. Our findings provide insight into consumer attention span based on eye-tracking and, thus, contribute to the discipline of attention-based marketing. These findings based on various responses to the retail environment provide useful information and design guidelines for retailers and designers, who are interested in improving the quality of retail design in consideration of interest of target customers in the retail environment.

This study offers practical insights for retailers and designers to formulate visual merchandising strategies that can positively influence consumer attention and emotion. The findings can assess cues utilized in the retail environments. Our study overcomes the limitations of self-reporting methods traditionally used to study consumer responses. The research findings imply that introducing new trends using visual merchandising elements, such as display fixtures and props, contributes to the creation of visual experiences in retail environments. This study further provides insight for designers, architects, and retailers on how customers engage with their retail environment and direct visual attention, in tandem with emotional arousal in response to visual stimuli. A consumer-centric approach could eventually increase consumer satisfaction and impact brand loyalty. When a retailer plans a new physical store, the designer can consider highlighting visual merchandising elements known to attract the attention of consumers and stimulate emotional arousal. The findings suggest a foundation for a theoretical framework to establish an evidence-based design process for enhancing retail environments.

We further demonstrated how, and to what extent, visual merchandising produces consumer responses based on collected biometrics. For example, in the case of the retail environment of the brand, the visual merchandising elements that caught the attention of subjects were facade, logo signage, seating in the service area, props such as planter boxes boxes at the entrance, and a large media screen on the central wall. The results also showed that the large media screen caught the attention of participants, provided useful information to learn about the latest products through advertising, and stimulated intellectual curiosity. Thus, the effects of visual merchandising elements can contribute to improving consumer interaction with the brand.

The experimental research methods using VR and eye-tracking suggested in this study can be used by architects and designers in designing retail environments. Of course, architects and designers plan a new store design based on their institutional knowledge and assumptions about how consumers may behave and respond to environments. However, prior to investing significant resources in the actual building process, VR simulations can be used as three-dimensional prototypes to obtain evidence on how consumers may respond psychologically by tracking their interest using biosignal sensors. The results of these VR-integrated eye-tracking experiments can be used by retailers to understand how different consumer segments engage with their environments and, thus, apportion their visual attention to visual elements.

This study has some limitations that affect the generalizability of the results. First, the primary limitation is the number of cases for the retail environment. Second, we had a limited number of subjects participated in the experiment. However, we focused on developing a conceptual framework and demonstrated the applicability of integrated eye-tracking VR experiments. The viability of the experimental protocol can be further investigated by replicating this study using different stimuli, such as an augmented reality (AR) store and online shopping experience. As personalized service is key in marketing, statistical analysis is required to understand individual differences in consumer attributes, such as among different age groups, cultural backgrounds, and education levels, and the responses thereof in future studies.

Future studies should be extended to test a wider range of brand-related stimuli (Brakus et al., 2009) to understand the conscious and unconscious reactions of consumers while interacting with the environmental design. The visual stimuli should consider retail environments to have a more diverse product mix and visually stimulate attention and arousal both to male and female consumers. Furthermore, it would be helpful to empirically investigate the relation between vision studies and purchase intention in the future study. Intangible elements that evoke behavioral responses could also be assessed to understand consumer experience in future research. For example, scholars can consider examining how background music or frangrance in retail environments influences the psychological responses and shopping behaviors of consumers. Thus, future studies should test different conditions of brand-related stimuli on consumer satisfaction and brand loyalty. We also recommend that scholars consider VR and AR simulations of new retail environments in online marketing, as these technologies are expected to revolutionize consumer experiences of retailing. For instance, three-dimensional virtual stores could be employed as experimental stimuli to measure how novel VR experience can influence consumer purchase decisions.

To quantify human psychological response in the designed elements, diverse biosensors can be employed to test the biosignals of participants to visual stimuli. For example, design physiology can be assessed through eye-tracking, electrodermal activity, heart rate variability, and facial emotional expression; and design neurocognition can be measured by electroencephalography, functional near infrared spectroscopy, and functional magnetic resonance imaging (Gero and Milovanovic, 2020). Furthermore, the multimodal study can investigate emotional quantities and their correlation with several physiological variables such as electroencephalogram, galvanic skin response, and eye-tracking. Thus, future research has the potential to generate more evidence to enhance retail environments by combining emerging technologies and a neuroscience approach. More challenges in attention-based marketing need to be explored to provide evidence-based insight; such inquiries will contribute to business development and success and, as a result, better adoption of strategies to affect consumer attention, emotion, and preferences.

## Data Availability Statement

The raw data supporting the conclusions of this article will be made available by the authors, without undue reservation.

## Ethics Statement

The studies involving human participants were reviewed and approved by Yonsei University IRB. The patients/participants provided their written informed consent to participate in this study. Written informed consent was obtained from the individual(s) for the publication of any potentially identifiable images or data included in this article.

## Author Contributions

NK contributed to the conception and design of the study, conducted the experiments and collected the data, and analyzed and interpreted the results under the supervision of HL. All authors contributed to the manuscript and approved it for publication.

## Conflict of Interest

The authors declare that the research was conducted in the absence of any commercial or financial relationships that could be construed as a potential conflict of interest.

## Publisher's Note

All claims expressed in this article are solely those of the authors and do not necessarily represent those of their affiliated organizations, or those of the publisher, the editors and the reviewers. Any product that may be evaluated in this article, or claim that may be made by its manufacturer, is not guaranteed or endorsed by the publisher.
